# Oral metastasis of renal cell carcinoma mimicking recurrence of excised malignant myoepithelioma: A case report

**DOI:** 10.3892/mco.2020.2204

**Published:** 2020-12-30

**Authors:** Yoshihiro Morita, Takaki Iwagami, Chisato Kawakita, Yukiko Kusuyama, Atsuko Niki-Yonekawa, Nobuo Morita

Mol Clin Oncol 9: 66-69, 2018; DOI: 10.3892/mco.2018.1630

Following the publication of this article, the authors have realized that, in the legend of Fig. 5 on p. 68, ‘NOS’, was incorrectly defined as ‘nitric oxide synthase’; the correct definition should have been given as ‘not otherwise specified’.

The Editor of *Molecular and Clinical Oncology* regrets that this error was introduced into the paper, and apologizes to the author and to the readership for any inconvenience caused.

